# Outpatient parenteral antimicrobial therapy delivery, readmission rates, and multidisciplinary teams: a scoping review of the impact of published quality indicators

**DOI:** 10.1017/ash.2026.10321

**Published:** 2026-03-03

**Authors:** Jackson Musuuza, Julie Keating, Meghan Brennan, Leslie Christensen, Charlie Wray, Marin L. Schweizer

**Affiliations:** 1 Department of Medicine, University of Wisconsin Madison, Madison, USA; 2 VA Medical Center Madison: William S Middleton Memorial Veterans Hospital, USA; 3 University of Wisconsin-Madison Ebling Library, USA; 4 Department of Medicine, University of California San Francisco, USA

## Abstract

**Background:**

Outpatient parenteral antimicrobial therapy (OPAT) reduces hospitalization, yet poor standardization and implementation contribute to readmission rates near 25%. The Infectious Diseases Society of America recommends structured and multidisciplinary OPAT programs. Twelve quality indicators, spanning organization, initiation, continuation, and outcome domains, have been proposed to improve OPAT delivery. Our scoping review assessed associations between reported OPAT quality indicators and patient readmission.

**Methods:**

We searched PubMed, Embase, Cochrane CENTRAL, Web of Science, and Google Scholar from database inception through May 1, 2025, for studies of adults discharged on OPAT, managed by multidisciplinary teams, and reporting readmission rates. Data included presence of each quality indicator, team composition, and readmission rates. Readmission was categorized as low (<10%) or high (≥10%).

**Results:**

Of 2,610 studies screened, 18 (5,027 patients) met criteria. The median readmission rate was 11.3 (IQR 8–20). All studies reported a structured OPAT program and formal OPAT team. Initial patient assessment by a competent team member was more common in studies with lower readmissions. Reporting more indicators (range 4–11) did not significantly correlate with fewer readmissions. Organization and initiation indicators were reported more frequently than continuation and outcome indicators. All programs included an infectious diseases physician; 94% included nurses, 55% pharmacists, 28% social workers, and 11% hospitalists.

**Conclusions:**

Higher quantity of reported indicators did not predict fewer readmissions. Future research should explore team engagement, including potential roles of hospitalists and social workers to strengthen care transitions, and the impact of continuation and outcome indicators on readmissions.

## Introduction

Certain severe infections such as osteomyelitis, endocarditis, and bloodstream infections routinely require extended hospitalization for intravenous antimicrobial treatment.^1^ However, extended hospitalizations strain the health care system, reduce bed availability, and increase the risk of adverse patient outcomes such as healthcare-associated infections and other adverse events such as falls.^2,3^ Therefore, for eligible patients, outpatient parenteral antimicrobial therapy (OPAT) is a pragmatic way to provide intravenous antimicrobials while reducing risks. Three models of OPAT delivery have been adapted, including the home model (via healthcare provider visit or self-administration), infusion centers, and skilled nursing facilities (SNF).^4^

Significant efforts continue to improve OPAT delivery and resulting patient outcomes. One approach recommended by the Infectious Diseases Society of America (IDSA) is the use of multidisciplinary teams to develop and manage OPAT programs.^5^ These teams could include infectious disease (ID) physicians, hospitalists, nurses, social workers, and pharmacists that are dedicated to the OPAT program.^5,6^ Studies show that patients discharged on OPAT with multidisciplinary care have better outcomes, including fewer readmissions.^7^

Despite these developments, poor standardization and implementation of OPAT delivery persist. To address this issue, Berrevoets et al. (2019) developed a set of quality indicators for OPAT that can be used as metrics for quality assessment and improvement.^8^ Using a RAND-modified Delphi procedure and review of the literature,12 quality indicators for optimal OPAT care were developed.^8^ The quality indicators were classified under 4 major domains: Organization, Initiation, Continuation, and Outcome (Table 1).

**Table 1.** Twelve OPAT quality indicators classified under 4 domains

Domain	Quality indicators
**Organization**	There should be a structured OPAT program to provide a framework for safe and effective care There should be a formal OPAT care team There should be a policy on patient selection criteria for OPAT
**Initiation**	There should be an OPAT treatment and monitoring plan A competent member of the OPAT team should perform the initial assessment Patients and their families should be informed about OPAT
**Continuation**	There should be a mechanism in place for urgent discussion and review of emergent clinical problems during OPAT according to clinical need There should be a system in place for rapid communication between the patient and team members Laboratory results should be delivered to physicians within 24 hours after obtaining material for testing
**Outcome**	The OPAT team should document clinical response to antimicrobial management The OPAT team should document adverse events related to devices, antibiotic use, and toxicity The OPAT team should monitor quality indicators for OPAT care and make this data available

OPAT, outpatient parenteral antimicrobial therapy.

Correlations between the frequency that these indicators are incorporated into OPAT programs and improved OPAT outcomes are unknown.

The primary purpose of this scoping review was to assess the association between the 12 quality indicators and readmission rates. The research question was: Is reporting use of more quality indicators associated with low readmission rates? The secondary purpose was to describe the composition of reported multidisciplinary teams in included studies. A scoping review was done because the quality indicators were relatively recently published. We did not anticipate that many studies had deliberately reported these indicators; thus, it was not possible to answer a more comparative question through a systematic review.

## Methods

### Study design

We conducted this scoping review following the methodological framework described by Arksey and O’Malley: (1) formulating the research question, (2) identifying relevant studies, (3) selecting eligible studies, (4) charting the data, and (5) collating, summarizing, and reporting the results.^9^ We followed the Preferred Reporting Items for Systematic Reviews and Meta-Analyses Extension for—Scoping Reviews (PRISMA-ScR) (online supplementary material).^10^ This review was registered with Open Science Framework on May 2, 2025 (https://doi.org/10.17605/OSF.IO/DP6XJ).

### Eligibility

The inclusion and exclusion criteria were developed using the Population–Concept–Context (PCC) framework.^11^

- Population: Studies involving hospitalized adult patients (≥18 years) who were discharged on parenteral antibiotics.
- Concept: The review focused on outpatient parenteral antibiotics and whether reporting of quality indicators was associated with readmission rates.
- Context: Discharge from acute care hospitals with no limitation to OPAT delivery models.

We included original studies from all study designs that met the following inclusion criteria: (1) reported or implied the presence of an OPAT multidisciplinary team, defined as two or more types of healthcare providers working on an OPAT program, and (2) reported OPAT-related outcomes, one of which must have been readmission rates. Studies were excluded if they were not written in English or were published abstracts only (including conference abstracts).

### Search strategy and study selection

We collaborated with a research librarian (LAC) to develop and execute a comprehensive search for literature related to OPAT care. A search was developed in PubMed and then translated into the following databases: Embase.com (Elsevier), Cochrane Central Register of Controlled Trials (CENTRAL) via Cochrane Library (Wiley), and Web of Science Core Collection (Clarivate) as a multi-file search of Science Citation Index-Expanded and Emerging Sources Citation Index. All searches were performed from database inception through May 1, 2025. In Embase, an inclusion filter was used to limit results to Embase only, and an exclusion filter was used to remove conference abstract from the results. No other filters were applied to the results. A Google Scholar search was executed on May 1, 2025, and the first 200 results, sorted by relevance, were exported. The complete search strategies are available in the Supplemental Material. Results were downloaded to a citation manager (EndNote) and underwent manual de-duplication by the research librarian. Unique records were uploaded to a web-based screening platform (Covidence) for independent review by team members.

Two reviewers (JSM, JAK) pilot tested the eligibility criteria, screened abstracts and full texts, and extracted data. The inclusion and exclusion criteria were pilot tested on 10 records. Reviewers independently screened the 10 abstracts and then met to discuss any discrepancies in the application of the eligibility criteria. After the pilot test, all studies were independently screened; conflicts were resolved through discussion between the two authors.^10^ A similar screening process was used for the full text review phase; all full texts were independently reviewed, with conflicts resolved in discussion between the two authors. No major discrepancies were identified between reviewers. Minor discrepancies such as differing assessments of whether a study involved a multidisciplinary team were resolved through discussion between the two reviewers.

### Data extraction and analysis

We developed a data extraction form *a priori,* then two reviewers (JSM and JAK) separately reviewed two included studies using the tool and discussed any variability to finalize the tool. These reviewers then independently extracted the data, with abstracted variables: first author, year of publication, country, type of evidence, study design, aim of publication, patient demographics, discharge to home/skilled nursing facility, sample size, readmission rate, multidisciplinary team composition by role, and reporting the presence of each of the twelve quality indicators. Extracted data were entered into an Excel spreadsheet. A descriptive analysis to summarize the data was conducted using Stata software, version 19.0 (Stata Corp. College Station, Texas). Statistical tests were two-tailed; a *P* value below .05 was considered significant. We categorized readmission rates as low (<10%) and high (≥10%), based on Centers for Medicare and Medicaid Services (CMS) data and published OPAT literature. CMS reports 30-day readmission rates for U.S. hospitals ranging from 10.1% to 19.1%, while studies of patients discharged on OPAT show rates as high as 26%.^7,12,13^

We calculated the frequency of reporting of each quality indicator and assessed readmission rates associated with each of the quality indicators reported. Further, we calculated the total number of quality indicators reported for each study, categorized these as few (6 or less) and many (7 or more), and assessed their association and readmission rates. Finally, we calculated the frequency of reporting specific professions (ID physician, pharmacist, registered nurse (RN), hospitalist, social worker, and others (eg, hematologist, orthopedic surgeons) for the individuals within multidisciplinary teams. Critical appraisal of individual sources of evidence was not performed as it is not required for scoping reviews. Additional team members (MB, CMW, MLS) contributed to interpretation of findings along with JSM and JAK.

## Results

### Characteristics of included studies

**Figure 1.**
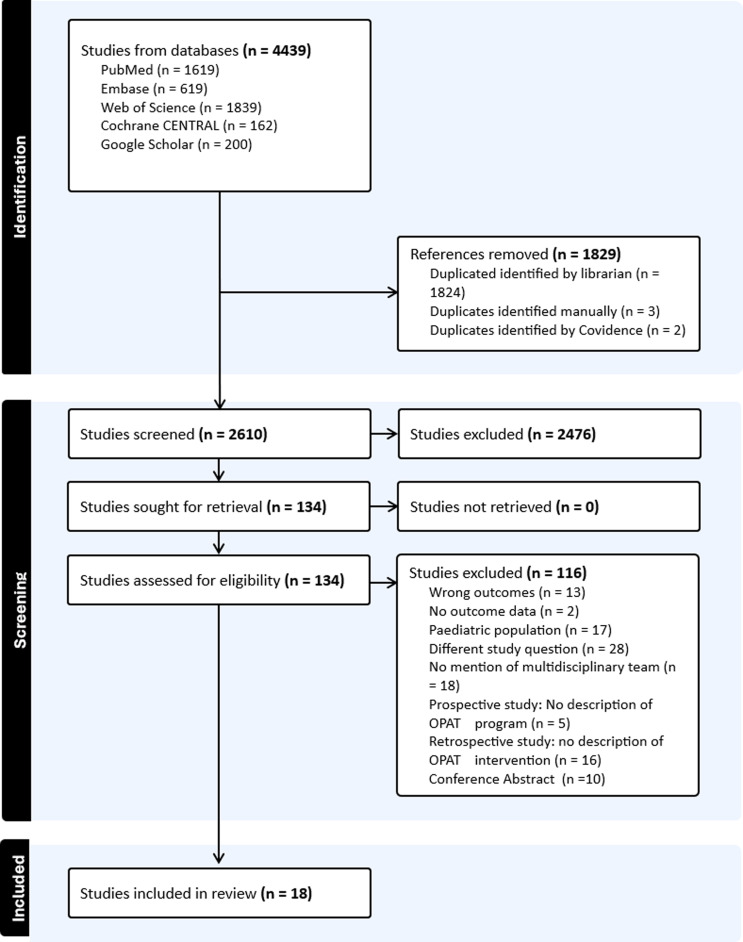
PRISMA flow diagram.

The search strategy identified 2,610 unique studies. Following title and abstract screening, 129 studies underwent full-text review, and 18 met eligibility criteria for inclusion in this review (Figure 1). The included studies examined a total of 5,027 patients.

Details about the characteristics of included studies were reported as online supplementary material. Here we provide the summarized findings. Fifty percent (9/18) of the studies were conducted in the United States (US), 16% (3/18) in Belgium, and one study each from Australia, Brazil, France, Norway, Saudi Arabia, and Sweden. In terms of study designs, 50% (9/18) were retrospective cohort studies, 44% (8/18) were prospective cohorts, and one was a quasi-experimental study. All studies reported on adult patient populations, and the majority of patients (61%) were male. All studies included patients discharged home; 6/18 (33%) of studies also included patients discharged to SNFs. Only five studies reported on the mortality outcome, for a total of 1% of patients (64/5704). The percentage of OPAT patients who were readmitted ranged from 0 to 27.9%, mean 11.3 (IQR 8–20). Most of the studies did not report data on multidrug-resistant organisms (MDRO) and for those that did 33% (6/18) provided aggregate data combining several MDROs.

### Quality indicators, readmission rates, and multidisciplinary teams

Total quality indicators reported by a study ranged from 4 to 11; no studies reported all 12 quality indicators. Most studies reported a total of 5 (4/18) or 8 (4/18) quality indicators. There was no association between readmission rates and presence of many (7 or more out of 12) quality indicators vs. few (6 or less) quality indicators (*P* = .64).

All studies reported that they had the quality indicators *Structured OPAT Program* and *Formal OPAT Care Team*. Eighty-three percent (15/18) of studies reported having a *Competent Member of the OPAT Team to Perform the Initial Assessment* and *Informing Patients and Their Families about OPAT*. The least reported quality indicators were *Delivering Results to Physicians in 24 Hours* (1/18) and *OPAT Team Monitoring for Quality Indicators for OPAT Care* (3/18).

The quality indicator *Competent Member of the OPAT Team to Perform the Initial Assessment* was more common in studies with lower readmissions although these results were not statistically significant. Only 3 studies reported having an *OPAT Team Member Monitoring Quality Indicators for OPAT Care and Making This Data Available*. These 3 studies were in the higher readmissions category (Table 2 and online supplementary material).

**Table 2.** Percent readmission and quality indicators reported by each study under the four domains: organization, initiation, continuation and outcome

First Author, Year	Organization	Initiation	Continuation	Outcome	Percent Readmission
Agnihotri 2023^7^	1. Structured OPAT program 2. Formal OPAT care team	1. Initial assessment by competent OPAT team member 2. Patient and family involvement	1. Mechanism for handling emergent issues	None	7
Bhava 2015^20^	1. Structured OPAT program 2. Formal OPAT care team 3. Policy on patient selection criteria for OPAT	1. OPAT treatment and monitoring plan 2. Initial assessment by competent OPAT team member 3. Patients and their families informed about OPAT	1. System for rapid communication between patient and care team	1. Documentation of clinical response 2. Documentation of adverse events	20
Bradle 2023^21^	1. Structured OPAT program 2. Formal OPAT care team 3. Policy on patient selection criteria for OPAT	1. OPAT treatment and monitoring plan	1. Mechanism for handling emergent issues	None	20
Brique 2020^22^	1. Structured OPAT program 2. Formal OPAT care team 3. Policy on patient selection criteria for OPAT	1. Initial assessment by competent OPAT team member 2. Patients and their families informed about OPAT	None	None	12
Heintz 2011^23^	1. Structured OPAT program 2. Formal OPAT care team	1. Initial assessment by competent OPAT team member	None	1. Documentation of clinical response 2. Documentation of adverse events	5
Ibaraki 2024^24^	1. Structured OPAT program 2. Formal OPAT care team	1. Patients and their families informed about OPAT	1. Mechanism for handling emergent issues 2. System for rapid communication between patient and care team	1. Documentation of clinical response 2. Documentation of adverse events 3. OPAT team monitored quality indicators for OPAT care and made this data available	20
Johansson 2001^25^	1. Structured OPAT program 2. Formal OPAT care team 3. Policy on patient selection criteria for OPAT	1. OPAT treatment and monitoring plan 2. Initial assessment by competent OPAT team member 3. Patients and their families informed about OPAT	1. System for rapid communication between patient and care team	None	0
Keller 2023^26^	1. Structured OPAT program 2. Formal OPAT care team	1. OPAT treatment and monitoring plan 2. Initial assessment by competent OPAT team member 3. Patients and their families informed about OPAT	1. Mechanism for handling emergent issues	None	27.9
Madali 2017^27^	1. Structured OPAT program 2. Formal OPAT care team 3. Policy on patient selection criteria for OPAT	1. OPAT treatment and monitoring plan 2. Initial assessment by competent OPAT team member 3. Patients and their families informed about OPAT	1. Mechanism for handling emergent issues 2. System for rapid communication between patient and care team	1. Documentation of clinical response 2. Documentation of adverse events 3. OPAT team monitored quality indicators for OPAT care and made this data available	13
Mansour 2018^28^	1. Structured OPAT program 2. Formal OPAT care team	1. OPAT treatment and monitoring plan 2. Initial assessment by competent OPAT team member 3. Patients and their families informed about OPAT	1. Mechanism for handling emergent issues 2. System for rapid communication between patient and care team	1. Documentation of clinical response 2. Documentation of adverse events	13
Missiaen 2024^29^	1. Structured OPAT program 2. Formal OPAT care team	1. OPAT treatment and monitoring plan	1. Mechanism for handling emergent issues	None	17
Oliveira 2015^30^	1. Structured OPAT program 2. Formal OPAT care team 3. Policy on patient selection criteria for OPAT	1. OPAT treatment and monitoring plan 2. Initial assessment by competent OPAT team member 3. Patients and their families informed about OPAT	1. Mechanism for handling emergent issues 2. System for rapid communication between patient and care team	1. Documentation of clinical response 2. Documentation of adverse events	3
Quinten2020^31^	1. Structured OPAT program 2. Formal OPAT care team 3. Policy on patient selection criteria for OPAT	1. OPAT treatment and monitoring plan 2. Initial assessment by competent OPAT team member 3. Patients and their families informed about OPAT	1. Mechanism for handling emergent issues 2. System for rapid communication between patient and care team	1. Documentation of clinical response 2. Documentation of adverse events	9
Rolland 2023^32^	1. Structured OPAT program 2. Formal OPAT care team 3. Policy on patient selection criteria for OPAT	1. OPAT treatment and monitoring plan 2. Initial assessment by competent OPAT team member 3. Patients and their families informed about OPAT	1. Mechanism for handling emergent issues	1. Documentation of clinical response 2. Documentation of adverse events	9
Skogen 2024^33^	1. Structured OPAT program 2. Formal OPAT care team 3. Policy on patient selection criteria for OPAT	1. OPAT treatment and monitoring plan 2. Initial assessment by competent OPAT team member 3. Patients and their families informed about OPAT	None	1. Documentation of clinical response 2. Documentation of adverse events	9
Tan 2017^34^	1. Structured OPAT program 2. Formal OPAT care team 3. Policy on patient selection criteria for OPAT	1. OPAT treatment and monitoring plan 2. Initial assessment by competent OPAT team member 3. Patients and their families informed about OPAT	None	1. Documentation of clinical response 2. Documentation of adverse events	8
Tan 2023^35^	1. Structured OPAT program 2. Formal OPAT care team 3. Policy on patient selection criteria for OPAT	1. OPAT treatment and monitoring plan 2. Initial assessment by competent OPAT team member 3. Patients and their families informed about OPAT	None	1. Documentation of clinical response 2. Documentation of adverse events	26
Zikri 2021^36^	1. Structured OPAT program 2. Formal OPAT care team 3. Policy on patient selection criteria for OPAT	1. OPAT treatment and monitoring plan 2. Initial assessment by competent OPAT team member 3. Patients and their families informed about OPAT	1. System for rapid communication between patient and care team 2. Laboratory results delivered to physicians within 24 hours after testing	1. Documentation of clinical response 2. Documentation of adverse events 3. OPAT team monitored quality indicators for OPAT care and made this data available	10.5

OPAT, outpatient parenteral antimicrobial therapy. SNF, Skilled Nursing Facility. MDROs, Multidrug-Resistant Organisms.

All studies reported having an ID physician as part of their multidisciplinary OPAT team; 16 (94%) had a registered nurse; 10 (55%) had a pharmacist; 5 (28%) had a social worker, and 2 (11%) had a hospitalist (Table 3). Seven studies reported having other professionals on the OPAT team who were either members of the hospital primary team that was treating the patient (e.g., a hematologist, urologist, or orthopedic surgeons) or individuals that only carried out clerical duties. Two studies that reported having a hospitalist on the OPAT team were classified as having high readmission rates.

**Table 3.** Reported members of multidisciplinary teams

First Author, Year	ID Physician	Hospitalist	RN	Pharmacist	Social worker	Other
Agnihotri 2023	Y	−	Y	−	−	−
Agnihotri 2023 ^7^	Y	−	Y	Y	Yes	Y(Unspecified staff in outpatient clinic, but formal OPAT team members)
Bhava 2015 ^20^	Y	−	Y	−	−	−
Bradle 2023 ^21^	Y	Y	Y	Y	Yes	Y(Orthopedic and urology surgery physicians, primary care physicians)
Brique 2020 ^22^	Y	−		Y	Yes	
Heintz 2011 ^23^	Y	−	Y	−	−	−
Ibaraki 2024 ^24^	Y	−	Y	Y	−	Y(Hematologist)
Johansson 2001 ^25^	Y	−	Y	Y	−	Y(Research assistant)
Keller 2023 ^26^	Y	−	Y	−	Yes	Y(Program administrator)
Madali 2017 ^27^	Y	−	Y	Y	−	Y(Clerical support person)
Mansour 2018 ^28^	Y	Y	−	Y	Yes	
Missiaen 2024 ^29^	Y	−	Y	−	−	−
Oliveira 2015 ^30^	Y	−	Y	Y	−	Y(Care delivery manager, microbiologist)
Quinten 2020 ^31^	Y	−	Y	−	−	−
Rolland 2023 ^32^	Y	−	Y	−	−	−
Skogen 2024 ^33^	Y	−	Y	−	−	−
Tan 2017 ^34^	Y	−	Y	Y	−	−
Tan 2023 ^35^	Y	−	Y	Y	−	−
Zikri 2021 ^36^	Y	−	Y	Y	−	−

ID, Infectious Disease. OPAT, Outpatient Parenteral Antibiotic Therapy. RN, Registered Nurse. Y: Yes, N: No.

## Discussion

This scoping review examined the association between published quality indicators for OPAT (Table 1) and readmission rates and described the composition of reported multidisciplinary teams. Across 18 studies involving over 5,000 patients, most OPAT programs had structured teams consistent with IDSA guidelines,^5^ though reporting of specific quality indicators varied. None of the studies reported all 12 priority quality indicators proposed by Berrevoets et al.,^8^ and there was no clear association between the total number of indicators reported and lower readmission rates. However, because of a lack of standardized reporting guidelines for OPAT programs, reporting of each quality indicator was dependent on author discretion and article/journal requirements (such as limited word count). Absence of quality indicator reporting in a study does not necessarily imply absence of the indicator in practice. However, this scoping review provides an initial overview of the prevalence of quality indicator reporting in published OPAT literature.

Interestingly, a greater number of reported quality indicators did not correlate with lower readmission rates. This may reflect heterogeneity in study designs, patient populations, and outcome definitions, as well as differences in implementation fidelity. It also highlights that the presence of quality indicators in program documentation may not equate to consistent real-world application. The quality and sustainability of implementation may ultimately determine patient outcomes more than the number of indicators formally reported.

Variation in outcome definitions further complicates interpretation. Some studies reported only infection-related readmissions, while others included all-cause readmissions, making cross-study comparisons challenging. Standardizing OPAT outcome definitions and reporting frameworks would facilitate more meaningful benchmarking and enable the identification of effective practices.

Organizational indicators, including having a structured OPAT program and a formal multidisciplinary team, were reported in all included studies (as expected given our inclusion criteria of OPAT programs having a multidisciplinary team). This aligns with the 2018 IDSA OPAT guidelines, which emphasize that OPAT should be delivered through a coordinated, structured program with defined oversight by an ID physician and clear communication pathways among clinicians, patients, and caregivers.^5^

All included studies described having an ID physician as part of the OPAT team, underscoring the central role of ID specialists in antimicrobial stewardship and program oversight. Most teams also included registered nurses (94%) and pharmacists (55%), both of whom are essential for daily coordination, drug monitoring, and patient education. However, few programs included social workers/case managers (28%) or hospitalists (11%). The absence of hospitalists is notable because they often play a key role in transitions of care, bridging inpatient and outpatient management.^14–16^ Their involvement could enhance communication at discharge, promote continuity of care, and address logistical challenges that contribute to readmission. In addition, increasing hospitalist involvement, for example by integration of OPAT into standard discharge workflows, could potentially lower the burden on ID specialists. Likewise, social workers are critical in assessing patient support systems, housing stability, and financial barriers—factors that directly affect OPAT adherence and safety. The IDSA guidelines recommend including case managers or social workers within the OPAT team to ensure comprehensive discharge planning and patient support.^5^ While reporting the presence of hospitalists or social workers was not correlated with low readmission rates in our analysis, other factors (such as patient population) may have impacted these rates as discussed above. While hospitalist and social worker involvement presents an opportunity to improve OPAT care, their roles are likely necessary but not sufficient to lower readmission rates.

Quality indicator reporting frequency varied between the domains (Table 2). Many studies reported quality indicators in the organization and initiation domains (Table 2), suggesting that most programs have strong initiation processes in place. *Structured OPAT programs* provide consistency in patient selection, antimicrobial monitoring, and follow-up and have been shown to reduce unplanned readmissions and complications. *Initial assessment by a competent team member* was associated with lower readmission rates. Additionally, most studies reported that patients and caregivers were informed about OPAT prior to its initiation. These findings reinforce the IDSA recommendation that all OPAT candidates undergo a formal multidisciplinary assessment of clinical stability, vascular access, and home environment before discharge.^5,17^ Structured education on therapy and catheter care ensures that patients and caregivers can identify early complications and contact the OPAT team promptly, thereby preventing avoidable hospital readmissions.

In contrast, continuation domain quality indicators such as r*apid communication of laboratory results* or u*rgent review of clinical problems*, were infrequently reported. Only one study described a system to ensure laboratory results were delivered to physicians within 24 h, and very few studies detailed processes for urgent clinical review. Because of lack of reporting of indicators in the Continuation domain, it is unknown whether mechanisms for ongoing monitoring and communication remain underdeveloped or just unreported. The IDSA guidelines explicitly recommend regular laboratory and clinical monitoring and readily available communication channels between patients and providers to address emerging issues during therapy.^5,18^ Failure to document or implement these practices could contribute to complications such as catheter-related infections or antibiotic toxicities, leading to higher readmission rates.

Likewise, few studies reported outcome domain quality indicators (Table 2). Only three studies mentioned any formal mechanism for ongoing quality assessment. This lack of outcome reporting limits benchmarking and quality improvement across institutions. The IDSA guidelines explicitly encourage programs to track and review key outcomes including treatment completion, readmissions, and adverse events to guide program development and ensure accountability.^5^ The limited documentation of such monitoring underscores a gap in translating these recommendations into practice.

### Study limitations

This review has several limitations. As a scoping review, it aimed to map available evidence rather than assess study quality or causality. Heterogeneity in study designs, populations (e.g., as studies with higher acuity patients might have higher readmissions regardless of how well the OPAT program is designed), and outcome definitions limited comparability and precluded quantitative synthesis. As discussed above, reporting bias is possible, as absence of indicator reporting may not reflect absence of practice since reporting of each quality indicators depends on author discretion. Additionally, most included studies originated from high-income countries, which may limit generalizability to resource-limited settings. Despite these limitations, the review provides important insights into current OPAT quality practices and highlights areas for future standardization and improvement.

Overall, our findings reinforce the IDSA recommendation that OPAT be delivered through a formalized multidisciplinary team operating within a structured framework.^5^ To optimize outcomes, programs should focus on four core elements: (1) establish a defined multidisciplinary structure with clear roles for ID physicians, nurses, pharmacists, and social workers; (2) conduct standardized predischarge assessments and education; (3) ensure rapid and efficient communication and monitoring systems to manage complications; and (4) systematically collect and report program metrics for continuous evaluation and quality improvement cycles.^8,19^

### Conclusion

In summary, OPAT programs frequently report adherence to Organization and Initiation domain quality indicators but less consistently to Continuation and Outcome indicators. Structured multidisciplinary programs that assessed patient eligibility for OPAT were associated with lower readmissions than those lacking these assessments. However, limited reporting of monitoring and quality outcomes, as well as underrepresentation of hospitalists and social workers, suggests that important opportunities remain for improvement. Future research should prioritize standardized reporting of quality indicators and outcome measures and evaluate how specific indicators influence patient safety and program efficiency. Strengthening multidisciplinary collaboration and systematic quality tracking will be essential to optimizing OPAT delivery and ensuring safe, effective, and equitable care for patients requiring long-term parenteral antimicrobial therapy outside the hospital setting.

## Supporting information

10.1017/ash.2026.10321.sm001 Musuuza et al. supplementary material 1 Musuuza et al. supplementary material

10.1017/ash.2026.10321.sm002 Musuuza et al. supplementary material 2 Musuuza et al. supplementary material

## Supplementary material

The supplementary material for this article can be found at https://doi.org/10.1017/ash.2026.10321.
