# Supplementary material for: Outpatient parenteral antimicrobial therapy delivery, readmission rates, and multidisciplinary teams: a scoping review of the impact of published quality indicators
Source: Antimicrob Steward Healthc Epidemiol. 2026 Mar 3;6(1):e56. doi: 10.1017/ash.2026.10321 (PMC12963781; doi:10.1017/ash.2026.10321)
Supplement: Musuuza et al. supplementary material 2 — Musuuza et al. supplementary material [file S2732494X26103210sup002.docx]

Project Lead: Jackson Musuuza, MD, PhD

[Jmusuuza@medicine.wisc.edu](mailto:Jmusuuza@medicine.wisc.edu) | ORCID ID: 0000-0001-9447-7659

Department of Medicine, UW Madison

Librarian: Leslie A. Christensen, MA-LIS
[leslie.christensen@wisc.edu](mailto:leslie.christensen@wisc.edu) | ORCID ID: 0000-0002-4040-5593
Ebling Library for the Health Sciences, University of Wisconsin – Madison

**Summary:**

A search was developed in PubMed and then translated into the following databases: Embase.com (Elsevier), Cochrane Central Register of Controlled Trials (CENTRAL) via Cochrane Library (Wiley), and Web of Science Core Collection (Clarivate) as a multi-file search of Science Citation Index-Expanded and Emerging Sources Citation Index. All searches were performed on May 1, 2025. In Embase, an inclusion filter was used to limit results to Embase only, and an exclusion filter was used to remove conference abstract from the results. No other filters were applied to the results. A Google Scholar search was executed on May 1, 2025, and the first 200 results, sorted by relevance, were exported.

| **Database searched** | **Date searched** | **Results** |
| --- | --- | --- |
| PubMed | 5/1/2025 | 1619 |
| Embase.com (Elsevier) | 5/1/2025 | 619 |
| Web of Science (Clarivate): SCI-Expanded, ESCI, | 5/1/2025 | 1839 |
| Cochrane CENTRAL | 5/1/2025 | 162 |
| Google Scholar | 5/1/2025 | 200 |
|  |  |  |
| Total | | 4439 |
| Total after librarian deduplication | | 2,615 |
| Total after Covidence deduplication | | 2,613 |

| **PubMed Line by Line** | | |
| --- | --- | --- |
|  | | |
| 1 | “Ambulatory Care”[mesh] |  |
| 2 | “Outpatients”[mesh] |  |
| 3 | #1 OR #2 |  |
| 4 | “”Anti-Bacterial Agents”[mesh] |  |
| 5 | “Anti-Infective Agents”[mesh] |  |
| 6 | #4 OR #5 |  |
| 7 | “Infusions, Parenteral”[mesh] |  |
| 8 | “Home Infusion Therapy”[mesh] |  |
| 9 | #7 OR #8 |  |
| 10 | #3 AND #6 AND #9 |  |
| 11 | OPAT[tiab] OR OPATs[tiab] OR HIAT[tiab] |  |
| 12 | "outpatient parenteral antibiotic"[tiab:~5] OR "outpatient parenteral antibiotics"[tiab:~5] OR “outpatient intravenous antibiotic”[tiab:~5] OR “outpatient intravenous antibiotics”[tiab:~5] OR “outpatient IV antibiotic”[tiab:~5] OR “outpatient IV antibiotics”[tiab:~5] OR “outpatient infusion antibiotic”[tiab:~5] OR “outpatient infusion antibiotics”[tiab:~5] OR “outpatient infusions antibiotic”[tiab:~5] OR “outpatient infusions antibiotics”[tiab:~5] OR “outpatients intravenous antibiotic”[tiab:~5] OR “outpatients intravenous antibiotics”[tiab:~5] OR “outpatients IV antibiotic”[tiab:~5] OR “outpatients IV antibiotics”[tiab:~5] OR “outpatients infusion antibiotic”[tiab:~5] OR “outpatients infusion antibiotics”[tiab:~5] OR “outpatients infusions antibiotic”[tiab:~5] OR “outpatients infusions antibiotics”[tiab:~5] |  |
| 13 | “home parenteral antibiotic”[tiab:~5] OR “home parenteral antibiotics”[tiab:~5] OR “home intravenous antibiotic”[tiab:~5] OR “home intravenous antibiotics”[tiab:~5] OR “home IV antibiotic”[tiab:~5] OR “home IV antibiotics”[tiab:~5] OR “home infusion antibiotic”[tiab:~5] OR “home infusion antibiotics”[tiab:~5] OR “home infusions antibiotic”[tiab:~5] OR “home infusions antibiotics”[tiab:~5] |  |
| 14 | “continuous outpatient antibiotic”[tiab:~5] OR “continuous outpatient antibiotics”[tiab:~5] OR “continuously outpatient antibiotic”[tiab:~5] OR “continuously outpatient antibiotics”[tiab:~5] |  |
| 15 | "outpatient parenteral antimicrobial"[tiab:~5] OR "outpatient parenteral antimicrobials"[tiab:~5] OR "outpatient parenteral anti-microbial"[tiab:~5] OR "outpatient parenteral anti-microbials"[tiab:~5] OR “outpatient intravenous antimicrobial”[tiab:~5] OR “outpatient intravenous antimicrobials”[tiab:~5] OR “outpatient intravenous anti-microbial”[tiab:~5] OR “outpatient intravenous anti-microbials”[tiab:~5] OR “outpatient IV antimicrobial”[tiab:~5] OR “outpatient IV antimicrobials”[tiab:~5] OR “outpatient IV anti-microbial”[tiab:~5] OR “outpatient IV anti-microbials”[tiab:~5] OR “outpatient infusion antimicrobial”[tiab:~5] OR “outpatient infusion antimicrobials”[tiab:~5] OR “outpatient infusions antimicrobial”[tiab:~5] OR “outpatient infusions antimicrobials”[tiab:~5] OR “outpatient infusion anti-microbial”[tiab:~5] OR “outpatient infusion anti-microbials”[tiab:~5] OR “outpatient infusions anti-microbial”[tiab:~5] OR “outpatient infusions anti-microbials”[tiab:~5] OR "outpatients parenteral antimicrobial"[tiab:~5] OR "outpatients parenteral antimicrobials"[tiab:~5] OR "outpatients parenteral anti-microbial"[tiab:~5] OR "outpatients parenteral anti-microbials"[tiab:~5] OR “outpatients intravenous antimicrobial”[tiab:~5] OR “outpatients intravenous antimicrobials”[tiab:~5] OR “outpatients intravenous anti-microbial”[tiab:~5] OR “outpatients intravenous anti-microbials”[tiab:~5] OR “outpatients IV antimicrobial”[tiab:~5] OR “outpatients IV antimicrobials”[tiab:~5] OR “outpatients IV anti-microbial”[tiab:~5] OR “outpatients IV anti-microbials”[tiab:~5] OR “outpatients infusion antimicrobial”[tiab:~5] OR “outpatients infusion antimicrobials”[tiab:~5] OR “outpatients infusions antimicrobial”[tiab:~5] OR “outpatients infusions antimicrobials”[tiab:~5] OR “outpatients infusion anti-microbial”[tiab:~5] OR “outpatients infusion anti-microbials”[tiab:~5] OR “outpatients infusions anti-microbial”[tiab:~5] OR “outpatients infusions anti-microbials”[tiab:~5] |  |
| 16 | “home intravenous antimicrobial”[tiab:~5] OR “home intravenous antimicrobial”[tiab:~5] OR “home intravenous anti-microbial”[tiab:~5] OR “home intravenous anti-microbials”[tiab:~5] OR “home IV antimicrobial”[tiab:~5] OR “home IV antimicrobial”[tiab:~5] OR “home IV anti-microbial”[tiab:~5] OR “home IV anti-microbials”[tiab:~5] OR “home infusion antimicrobial”[tiab:~5] OR “home infusion antimicrobials”[tiab:~5] OR “home infusions antimicrobial”[tiab:~5] OR “home infusions antimicrobials”[tiab:~5] OR “home infusion anti-microbial”[tiab:~5] OR “home infusion anti-microbials”[tiab:~5] OR “home infusions anti-microbial”[tiab:~5] OR “home infusions anti-microbials”[tiab:~5] |  |
| 17 | “continuous outpatient antimicrobial”[tiab:~5] OR “continuous outpatient antimicrobials”[tiab:~5] OR “continuous outpatient anti-microbial”[tiab:~5] OR “continuous outpatient anti-microbials”[tiab:~5] OR “continuously outpatient antibiotic”[tiab:~5] OR “continuously outpatient antibiotics”[tiab:~5] OR “continuously outpatient anti-microbial”[tiab:~5] OR “continuously outpatient anti-microbials”[tiab:~5] OR “continuous outpatients antimicrobial”[tiab:~5] OR “continuous outpatients antimicrobials”[tiab:~5] OR “continuous outpatients anti-microbial”[tiab:~5] OR “continuous outpatients anti-microbials”[tiab:~5] OR “continuously outpatients antibiotic”[tiab:~5] OR “continuously outpatients antibiotics”[tiab:~5] OR “continuously outpatients anti-microbial”[tiab:~5] OR “continuously outpatients anti-microbials”[tiab:~5] |  |
| 18 | #11 OR #12 OR #13 OR #14 OR #15 OR #16 OR #17 |  |
| 19 | #10 AND #18 | 1617 |

---

**PubMed Search**
1619 Results

(((“Ambulatory Care”[mesh] OR “Outpatients”[mesh]) AND (“Anti-Infective Agents”[mesh]) AND ( “Infusions, Parenteral”[mesh] OR “Home Infusion Therapy”[mesh])) OR

(OPAT[tiab] OR OPATs[tiab] OR COPAT[tiab] OR HIAT[tiab] OR HIATs[tiab] OR "outpatient parenteral antibiotic"[tiab:~5] OR "outpatient parenteral antibiotics"[tiab:~5] OR “outpatient intravenous antibiotic”[tiab:~5] OR “outpatient intravenous antibiotics”[tiab:~5] OR “outpatient IV antibiotic”[tiab:~5] OR “outpatient IV antibiotics”[tiab:~5] OR “outpatient infusion antibiotic”[tiab:~5] OR “outpatient infusion antibiotics”[tiab:~5] OR “outpatient infusions antibiotic”[tiab:~5] OR “outpatient infusions antibiotics”[tiab:~5] OR “outpatients intravenous antibiotic”[tiab:~5] OR “outpatients intravenous antibiotics”[tiab:~5] OR “outpatients IV antibiotic”[tiab:~5] OR “outpatients IV antibiotics”[tiab:~5] OR “outpatients infusion antibiotic”[tiab:~5] OR “outpatients infusion antibiotics”[tiab:~5] OR “outpatients infusions antibiotic”[tiab:~5] OR “outpatients infusions antibiotics”[tiab:~5] OR “home parenteral antibiotic”[tiab:~5] OR “home parenteral antibiotics”[tiab:~5] OR “home intravenous antibiotic”[tiab:~5] OR “home intravenous antibiotics”[tiab:~5] OR “home IV antibiotic”[tiab:~5] OR “home IV antibiotics”[tiab:~5] OR “home infusion antibiotic”[tiab:~5] OR “home infusion antibiotics”[tiab:~5] OR “home infusions antibiotic”[tiab:~5] OR “home infusions antibiotics”[tiab:~5] OR "outpatient parenteral antimicrobial"[tiab:~5] OR "outpatient parenteral antimicrobials"[tiab:~5] OR "outpatient parenteral anti-microbial"[tiab:~5] OR "outpatient parenteral anti-microbials"[tiab:~5] OR “outpatient intravenous antimicrobial”[tiab:~5] OR “outpatient intravenous antimicrobials”[tiab:~5] OR “outpatient intravenous anti-microbial”[tiab:~5] OR “outpatient intravenous anti-microbials”[tiab:~5] OR “outpatient IV antimicrobial”[tiab:~5] OR “outpatient IV antimicrobials”[tiab:~5] OR “outpatient IV anti-microbial”[tiab:~5] OR “outpatient IV anti-microbials”[tiab:~5] OR “outpatient infusion antimicrobial”[tiab:~5] OR “outpatient infusion antimicrobials”[tiab:~5] OR “outpatient infusions antimicrobial”[tiab:~5] OR “outpatient infusions antimicrobials”[tiab:~5] OR “outpatient infusion anti-microbial”[tiab:~5] OR “outpatient infusion anti-microbials”[tiab:~5] OR “outpatient infusions anti-microbial”[tiab:~5] OR “outpatient infusions anti-microbials”[tiab:~5] OR "outpatients parenteral antimicrobial"[tiab:~5] OR "outpatients parenteral antimicrobials"[tiab:~5] OR "outpatients parenteral anti-microbial"[tiab:~5] OR "outpatients parenteral anti-microbials"[tiab:~5] OR “outpatients intravenous antimicrobial”[tiab:~5] OR “outpatients intravenous antimicrobials”[tiab:~5] OR “outpatients intravenous anti-microbial”[tiab:~5] OR “outpatients intravenous anti-microbials”[tiab:~5] OR “outpatients IV antimicrobial”[tiab:~5] OR “outpatients IV antimicrobials”[tiab:~5] OR “outpatients IV anti-microbial”[tiab:~5] OR “outpatients IV anti-microbials”[tiab:~5] OR “outpatients infusion antimicrobial”[tiab:~5] OR “outpatients infusion antimicrobials”[tiab:~5] OR “outpatients infusions antimicrobial”[tiab:~5] OR “outpatients infusions antimicrobials”[tiab:~5] OR “outpatients infusion anti-microbial”[tiab:~5] OR “outpatients infusion anti-microbials”[tiab:~5] OR “outpatients infusions anti-microbial”[tiab:~5] OR “outpatients infusions anti-microbials”[tiab:~5] OR “home intravenous antimicrobial”[tiab:~5] OR “home intravenous antimicrobial”[tiab:~5] OR “home intravenous anti-microbial”[tiab:~5] OR “home intravenous anti-microbials”[tiab:~5] OR “home IV antimicrobial”[tiab:~5] OR “home IV antimicrobial”[tiab:~5] OR “home IV anti-microbial”[tiab:~5] OR “home IV anti-microbials”[tiab:~5] OR “home infusion antimicrobial”[tiab:~5] OR “home infusion antimicrobials”[tiab:~5] OR “home infusions antimicrobial”[tiab:~5] OR “home infusions antimicrobials”[tiab:~5] OR “home infusion anti-microbial”[tiab:~5] OR “home infusion anti-microbials”[tiab:~5] OR “home infusions anti-microbial”[tiab:~5] OR “home infusions anti-microbials”[tiab:~5] OR “continuous outpatient antimicrobial”[tiab:~5] OR “continuous outpatient antimicrobials”[tiab:~5] OR “continuous outpatient anti-microbial”[tiab:~5] OR “continuous outpatient anti-microbials”[tiab:~5] OR “continuously outpatient antibiotic”[tiab:~5] OR “continuously outpatient antibiotics”[tiab:~5] OR “continuously outpatient anti-microbial”[tiab:~5] OR “continuously outpatient anti-microbials”[tiab:~5] OR “continuous outpatients antimicrobial”[tiab:~5] OR “continuous outpatients antimicrobials”[tiab:~5] OR “continuous outpatients anti-microbial”[tiab:~5] OR “continuous outpatients anti-microbials”[tiab:~5] OR “continuously outpatients antibiotic”[tiab:~5] OR “continuously outpatients antibiotics”[tiab:~5] OR “continuously outpatients anti-microbial”[tiab:~5] OR “continuously outpatients anti-microbials”[tiab:~5]))

---

**Embase.com
619 Results**

((‘outpatient care’/exp OR ‘outpatient’/exp OR ‘ambulatory care’/exp) AND (‘anti-infective therapy’/exp OR ‘antiinfective agent’/exp) AND (‘home intravenous therapy’/exp OR ‘infusion therapy’/exp OR ‘home infusion therapy’/exp OR ‘parenteral drug administration’/exp)) OR ( ((OPAT$ OR COPAT$ OR HIAT$ OR ((outpatient* OR home*) NEAR/5 (parenteral* OR intravenous* OR IV$ OR infusion* OR continuous* ) NEAR/5 (antibiotic* OR antimicrobial* OR anti-microbial*))):ti,ab,kw)) NOT ‘conference abstract’ AND ([embase]/lim NOT ([embase]/lim AND [medline]/lim) OR ([embase classic]/lim NOT ([embase classic]/lim AND [medline]/lim)))

---

**Web of Science**
1839 Results
 (TS=(OPAT$ OR COPAT$ OR HIAT$ OR ((outpatient* OR home*) NEAR/5 (parenteral* OR intravenous* OR IV OR IVs OR infusion* OR continuous* ) NEAR/5 (antibiotic* OR antimicrobial* OR anti-microbial*))))

---

**Cochrane CENTRAL**
162 Results
( ((OPAT$ OR COPAT$ OR HIAT$ OR ((outpatient* OR home*) NEAR/5 (parenteral* OR intravenous* OR IV$ OR infusion* OR continuous* ) NEAR/5 (antibiotic* OR antimicrobial* OR anti-microbial*))):ti,ab,kw))

--

**Google Scholar**200 results, sorted by relevancy
OPAT|OPAT|COPAT|COPAT|“outpatient parenteral antibiotic|”outpatient parenteral antimicrobial”|”outpatient intravenous antibiotic”
|”outpatient infusion antibiotic”|”outpatient intravenous antimicrobial”|”outpatient infusion antimicrobial”
